# Histone Demethylase PHF8 Is Required for the Development of the Zebrafish Inner Ear and Posterior Lateral Line

**DOI:** 10.3389/fcell.2020.566504

**Published:** 2020-11-23

**Authors:** Jing He, Zhiwei Zheng, Xianyang Luo, Yongjun Hong, Wenling Su, Chengfu Cai

**Affiliations:** ^1^Department of Otorhinolaryngology, Zhongshan Hospital of Xiamen, School of Medicine, Xiamen University, Xiamen, China; ^2^Department of Otorhinolaryngology, Head and Neck Surgery, The First Affiliated Hospital, School of Medicine, Xiamen University, Xiamen, China; ^3^Teaching Hospital of Fujian Medical University, Xiamen, China; ^4^Xiamen Key Laboratory of Otolaryngology, Head and Neck Surgery, Xiamen, China

**Keywords:** PHF8, inner ear, posterior lateral line, zebrafish, organogenesis

## Abstract

Histone demethylase PHF8 is crucial for multiple developmental processes, and hence, the awareness of its function in developing auditory organs needs to be increased. Using *in situ* hybridization (ISH) labeling, the mRNA expression of *PHF8* in the zebrafish lateral line system and otic vesicle was monitored. The knockdown of PHF8 by morpholino significantly disrupted the development of the posterior lateral line system, which impacted cell migration and decreased the number of lateral line neuromasts. The knockdown of PHF8 also resulted in severe malformation of the semicircular canal and otoliths in terms of size, quantity, and position during the inner ear development. The loss of function of PHF8 also induced a defective differentiation in sensory hair cells in both lateral line neuromasts and the inner ear. ISH analysis of embryos that lacked PHF8 showed alterations in the expression of many target genes of several signaling pathways concerning cell migration and deposition, including the Wnt and FGF pathways. In summary, the current findings established PHF8 as a novel epigenetic element in developing auditory organs, rendering it a potential candidate for hearing loss therapy.

## Introduction

A primary cause of hearing loss is the abnormal development of inner ear structure, which is a highly dynamic process that includes modeling, cellular growth, migration, and differentiation. Although several regulators concerning the inner ear’s development have been the subject of studies, knowledge of the underlying molecular mechanisms is still incomplete. Nonetheless, epigenetic mechanisms, such as histone modifications, are essential in the development process of the auditory system (He et al., 2013, 2014, 2016a,b, 2020). For instance, it is believed that the histone deacetylases (HDACs) act as transcriptional regulators, modulating the differentiation and proliferation of hair cells during auditory organogenesis in zebrafish (He et al., 2016b). Accumulating evidence has proved that HDAC activity is pharmacologically inhibited through trichostatin A, which impedes cell proliferation, and thereby decreases the number of hair cells and supporting cells (He et al., 2014, 2016b). Other studies have shown that HDAC1, a novel function, regulates the morphogenesis of the inner ear of zebrafish (He et al., 2016a), as the knockdown of HDAC1 would alter the size, otolith formation and semicircular development of the inner ear, and induce a decline in the number of hair cells (He et al., 2016a). Thus, a complete understanding of the epigenetic changes in the development, physiology, and pathology of hearing would be of great importance, with potential applications in preventing and treating hearing loss in humans.

Plant homeodomain finger protein 8 (PHF8) is an X-related histone demethylase that contains a plant homeodomain and a catalytic Jumonji C (JMJC) domain with specificity for repressive modifications (Qi et al., 2010; Sun et al., 2015; Gu et al., 2016). Recently, the dysregulation of PHF8 has been linked to the inner ear’s aberrant development and diseases. For example, PHF8 mutations have been identified in a subset of patients with X-related intellectual disability and are often accompanied by cleaved lips/palate (Laumonnier et al., 2005; Abidi et al., 2007; Koivisto et al., 2007). The genetic silencing of PHF8 in a culture of cells results in postponed G1-S transition throughout the cell cycle development and damaged neuronal differentiation (Liu et al., 2010; Qiu et al., 2010). Additionally, *in vivo* functional studies have discovered that the morpholino (MO)-mediated knockdown of PHF8 leads to brain and craniofacial defects throughout zebrafish development (Qi et al., 2010) while retardation of a PHF8 homolog in *Caenorhabditis elegans* results in aberrant behavior, such as defective locomotion (Kleine-Kohlbrecher et al., 2010). Despite the evidence provided by these studies that PHF8 plays a potential role in the regulation of cell differentiation and survival during embryonic progression and adult behavior, the function of this demethylase in auditory organ development remains unexplored.

The zebrafish (*Danio rerio*) has an *in vivo* vertebrate system that provides an excellent model for exploring the functions of epigenetic mediators in multiple organogenesis such as the inner ear and diseases (Whitfield, 2002; He et al., 2017). The inner ear and the lateral line system constitute the zebrafish’s sensory organs that detect its surroundings underwater. Otoliths, semicircular canals, and five sensory patches constitute the structure of the representative vertebral inner ear in zebrafish (Bever and Fekete, 2002). In the embryo of zebrafish, two maculae are contained in each otic vesicle: the anterior utriculus plays a vestibular role, and the posterior sense macula (saccule) functions in hearing. Each macula is covered by an otolith attached to a kinocilium of hair cells. The lateral line system progresses from two placodes anterior and posterior to the otic vesicle through the FGF and Wnt signaling pathways via mutual interaction (Ghysen and Dambly-Chaudiere, 2004; Aman and Piotrowski, 2008; Ma and Raible, 2009). The system consists of discrete sensory organs termed as “neuromasts” that are settled in a specified mode on the body’s surface and respond to the currents of water (Nunez et al., 2009). Zebrafish are endowed with hair cells and supporting cells in both the inner ear and lateral line neuromasts, showing similarities to mammals in terms of morphology, function, and inner ear hair cells (Nicolson, 2005). Therefore, this model was employed to elucidate the role of PHF8 in the development of hearing.

According to the analysis of whole-mount *in situ* hybridization (WISH) in the embryonic period of zebrafish, a conclusion may be drawn that *PHF8* messenger RNA (mRNA) is expressed during the progression of mechanosensory organs, which includes the lateral line primordium, deposited neuromasts, and otic vesicle. The function of PHF8 in auditory organ development was evaluated by knockdown of the gene in zebrafish through MO injection. Furthermore, the injection of PHF8 antisense MO oligonucleotides reduced the number of neuromasts and developmental defects of the otoliths and semicircular canals in the inner ear. In contrast, the gene expression analysis of *PHF8* morphants revealed that the impact of PHF8 on the lateral line system was effectuated via Wnt and FGF pathways. Taken together, this study demonstrated that PHF8 is crucial for the development of the lateral line neuromasts and the inner ear for hearing normality in zebrafish.

## Materials and Methods

### Zebrafish Strains

Standard conditions were provided for maintaining the zebrafish. Hours post-fertilization (hpf) or days post-fertilization (dpf) were used to describe the duration of the existence of embryos and larvae. To prevent the formation of the staining complex, fish water with 0.003% 1-phenyl-2-thiourea (PTU; Sigma-Aldrich, St. Louis, MO, United States) was prepared for the treatment of embryos >14 hpf.

### MO Injection and mRNA Rescue

The MO oligomer (MO) targeting PHF8 was obtained from Gene Tools (Philomatch, OR, United States) and solubilized in germ-free water at a stock concentration of 1 mM. The embryos were injected with 8 ng PHF8 MO at the 1–2 cell stage. PHF8-MO was 5′-CAGTAAACCGGAACAGATGCCATTC-3′, *p53*-MO: 5′-GCGCCATTGCTTTGCAAGAATTG-3′, and the standard control MO was 5′-CCTCTTACCTCAGTTACAATTTATA-3′. To rescue the embryos injected with PHF8-MO, according to the instructions of the manufacturer, full-length complementary DNA (cDNA) that encodes zebrafish *PHF8* in pCS2 vector was utilized as the template for the synthesis of capped RNA. An equivalent of 150 pg of *PHF8* mRNA was injected for rescue experiments.

### Whole-Mount ISH

Digoxigenin-labeled RNA probes were synthesized as recommended by the manufacturer (Roche, Mannheim). Whole-mount ISH was conducted as described previously (Thisse and Thisse, 2008).

### Immunolabeling and Image Processing

Green fluorescent protein (GFP) (1:1000 dilution; Abcam, Cambridge, United Kingdom) and Sox2 (1:1000 dilution; Abcam) were used for immunohistochemistry. Briefly, the embryos were fixed in 4% paraformaldehyde (PFA) for 2 h at room temperature and permeabilized with phosphate-buffered saline (PBS) containing 0.5% Triton X-100 (PBT-2) for 0.5 h, followed by incubation in blocking solution (10% serum of a newborn donkey) for 1 h and incubation with antibodies overnight at 4°C. The images were processed by Adobe Photoshop software.

### BrdU Incorporation and Analysis

The embryos were dechorionated and incubated in 10 mM 5-bromo-2-deoxyuridine (BrdU; Sigma–Aldrich) at 48 hpf for 1 day at the temperature of 28.5°C. Immunocytochemistry detected BrdU inclusion. The larvae were anesthetized, fixed with 4% PFA for 2 h at room temperature in 0.02% MS-222 (ethyl 3-aminobenzoate methanesulfonate; Sigma–Aldrich), rinsed three times in PBT-2, and placed in 2 N HCl for 30 min at 37°C. Subsequently, the non-specific binding on the larvae was blocked for 1 h at room temperature, followed by incubation with monoclonal mouse BrdU antibody (1:200; Santa Cruz, Dallas, TX, United States; Cat. no. sc-32323) overnight at 4°C and secondary antibody for 1 h at 37°C before images were acquired.

### RT-PCR

Total RNA was isolated using the TRIzol Reagent (Invitrogen Life Technologies). The quantity and quality of RNA were assessed using a NanoDrop spectrophotometer (Thermo Scientific). First-strand cDNA was synthesized with the SuperScript III First-strand synthesis System.

### FM1-43FX Labeling

The operational hair cells in lateral line neuromasts were visualized using an indispensable stain FM1-43FX (Molecular Probes, Eugene, OR, United States) at a concentration of 3 μM to cultivate 3 dpf larvae for 45 s in the dark. After three washes with fresh water, the larvae were placed under anesthesia in 0.02% MS-222 and fixed with 4% PFA in PBS for 2 h at room temperature or 4°C overnight.

### Statistical Analysis

The statistical analysis was conducted using GraphPad Prism (version 6). Multiple comparisons adopted the one-way analysis of variance (ANOVA), while single comparisons employed a two-tailed Student’s *t*-test. All statistical data are presented as the mean ± standard error of the mean (SEM). A *p*-value of <0.05 indicated statistical significance.

## Results

### Expression Model of Phf8 During Zebrafish Organogenesis

**FIGURE 1:**
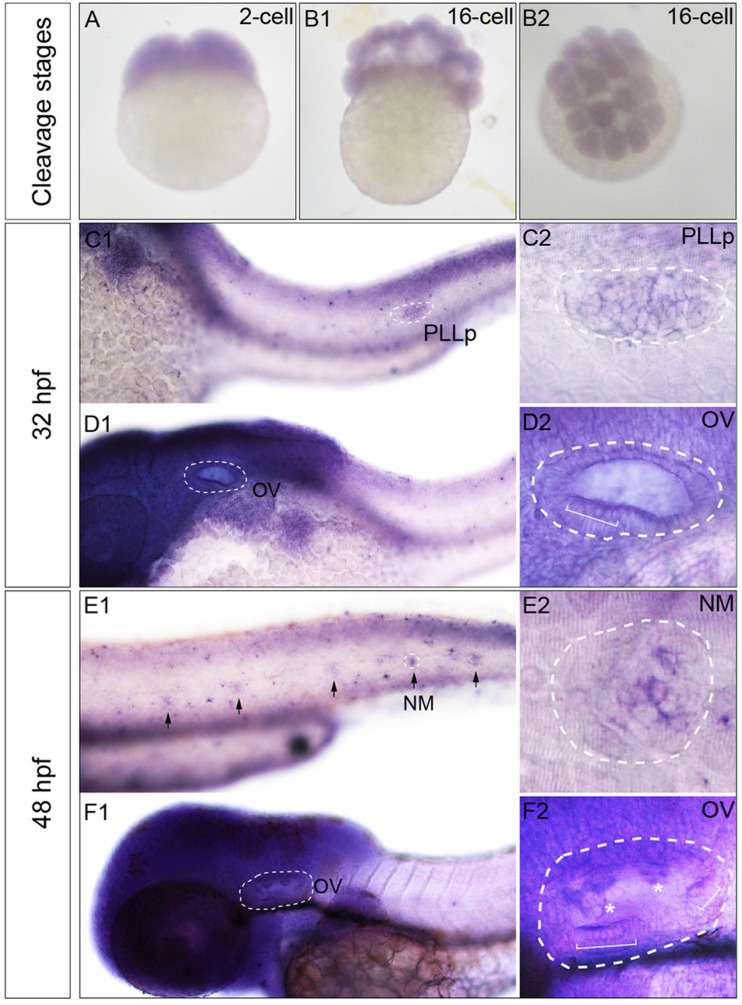
Transcription patterns of *Phf8* from 2-cell stage to 48 hpf. **(A,B)** *Phf8* expression was detected in both blastomeres at the 2-cell stage and in whole blastoderm at 16-cell stage. **(C–F)** Expression of *Phf8* in PLL system and otic vesicle at 32 and 48 hpf. (**C1,C2**; PLLp, 32 hpf), neuromasts (**E1,E2**; NM, 48 hpf), and otic vesicle (**D1,D2**,**F1,F2**; OV, 32 and 48 hpf). Black arrows indicate the deposited neuromast, the white asterisks show protrusions of the semicircular canal, and the white square bracket shows the cristae. PLL primordium, PLLp; Neuromast, NM; otic vesicle, OV. Arrows.

The expression pattern of zebrafish *Phf8* from the two-cell stage to 48 hpf were analyzed. Whole-mount ISH statistics suggested that *Phf8* expression was detected in the whole blastomeres in cleavage stages (two-cell stage, Figure 1A; 16-cell stage, Figures 1B1,B2). During development, *Phf8* was found to be mainly concentrated in the head region. In addition to the head region, the larvae showed *Phf8* staining in the migrating posterior lateral line (pLL) primordium by 32 hpf, (Figures 1C1,C2). *Phf8* was also detected in the otic vesicle, and staining was significant in the anterior domain (Figures 1D1,D2). At 48 hpf, *Phf8* was perceived in pLL neuromasts (Figure 1E1) and is highly expressed in the inner ear (Figure 1F1), which includes the semicircular canal and cristae (Figure 1F2). Inside the neuromast, predominant transcription of *Phf8* was effectuated in the central cluster cells of neuromasts (Figure 1E2). These results indicated that the expression pattern of *Phf8* is associated with the progression in the development of the hearing organs in zebrafish.

### PHF8 Is Required for pLL Morphogenesis

**FIGURE 2:**
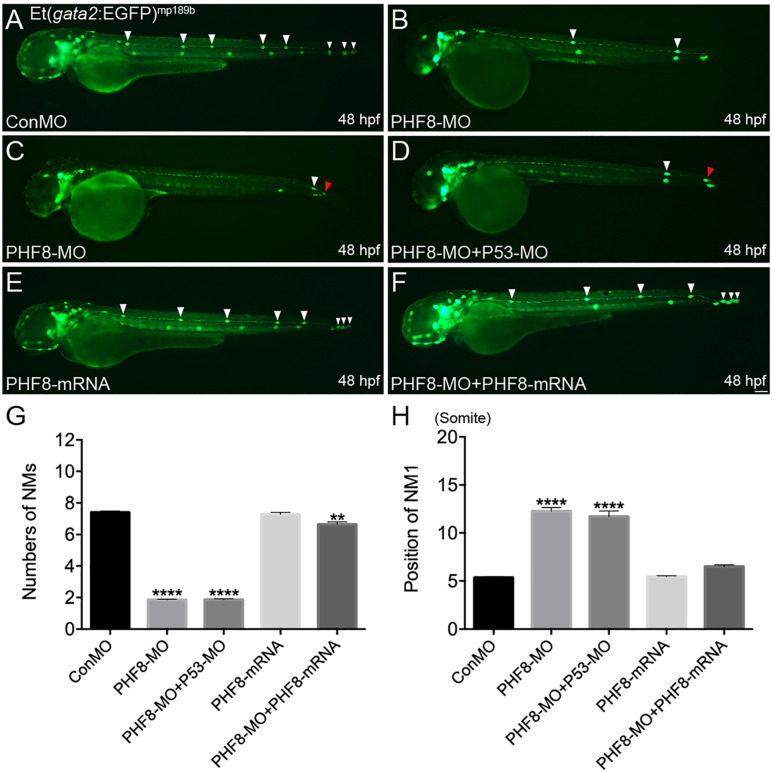
Abnormal PLL neuromast deposition in the PHF8 deficient embryos. **(A–F)** Optical appearance of fluorescence of 48 hpf larvae expressing Et(*gata2*:EGFP)^mp189b^. **(A)** The standard control MO (ConMO)-injected embryo illustrates a completed PLL mode. **(B–C)** PHF8-deficient embryos demonstrate a decrease in deposited neuromasts. **(D)** The embryo co-injected with PHF8-MO and p53-MO. **(E)** The embryo is injected with PHF8-mRNA. **(F)** The embryo co-injected with PHF8-MO and PHF8-mRNA. White arrowheads implicate the positions of the neuromasts on one side, Red arrowhead implicates the location of PLL primordium. **(G,H)** The quantity of PLL neuromasts **(G)** and the position of the first neuromast (NM1; **H**) were measured. Control embryos (ConMO; *n* = 112 embryos), PHF8 morphants (PHF8-MO; *n* = 319 embryos), PHF8-MO co-injected with P53-MO embryos (PHF8-MO + P53-MO; *n* = 115 embryos), PHF8 embryos injected with mRNA (PHF8-mRNA; *n* = 76 embryos), and PHF8-MO co-injected with PHF8-mRNA embryos (PHF8-MO + PHF8-mRNA; *n* = 80 embryos). **p < 0.01,****p < 0.0001 vs. the ConMO group. Data are presented as means ± SEM. All pictures showed lateral view, anterior left. Scale bars, 100 μm. Neuromast, NM.

*Phf8* is highly expressed in the brain and the lateral line system of the zebrafish during embryonic development. Thus, it was hypothesized that PHF8 functions were correlated to pLL morphogenesis and hair cells besides its function during the central nervous system’s development. Herein, we studied the role of PHF8 in the development of pLL system in the zebrafish embryos by downregulating its expression. Antisense MO oligo (PHF8-MO) was injected into the embryos at the one-to-two-cell stage to inhibit the expression of PHF8. The efficiency of the MO was confirmed by RT-PCR (Supplementary Figure 1A). In order to elucidate whether PHF8 functions in the formation of pLL, PHF8-MO was injected into transgenic Et(*gata2*:EGFP)^*mp*189*b*^ zebrafish expressing GFP in the pLL primordium and neuromast cells. The expression of GFP was inspected at 48 hpf, a stage at which the pLL primordium usually prevents migration at the tip of the tail, and neuromasts are deposited in a specific mode along the horizontal myoseptum. The downregulation of PHF8 with the MO did not cause any obvious gross morphological defects as compared to the control MO (ConMO) (Supplementary Figure 1B). The number of GFP-positive neuromasts formed by 48 hpf was significantly reduced in PHF8-MO-injected embryos. As shown in Figure 2A, 100% (112/112) of the ConMO-injected embryos demonstrated the regular mode of 7.4 ± 0.11 *gata2*-positive neuromasts at 48 hpf; however, in PHF8 morphants (8 ng of PHF8-MO) (*n* = 319) the average number was less (mean: 1.9 ± 0.04 neuromasts) compared to that of Con-MO. The majority of the embryos injected with PHF8-MO had one or two neuromasts (Figures 2B,C). The locations of the neuromasts were also disrupted in PHF8 morphants. The deposition of the first pLL neuromast (NM1) in the PHF8-deficient embryos posteriorly was delayed (Figure 2H). In order to exclude the off-target impact of p53-stimulated MO, the embryos (*n* = 115) were co-injected with p53-MO and PHF8-MO. The embryos with co-injection had fewer neuromasts than controls (mean: 1.8 ± 0.06 neuromasts, Figure 2D), implying that the deficiencies are not triggered by p53-induced apoptosis. The injection of *PHF8* mRNA did not induce excessive neuromasts (*n* = 76) (mean: 7.3 ± 0.13 neuromasts, Figures 2E,G), but when Et(*gata2*:EGFP)^*mp*189*b*^ embryos were co-injected with *PHF8* mRNA and PHF8-MO, the neuromast quantity was restored to 5.6 ± 0.12 (*n* = 80) (Figures 2F,G), implying a precise function of PHF8 in pLL formation. The statistical analysis showed that PHF8 functions included the quantity and localization control of neuromasts in the lateral lines.

### PHF8 Is Necessary for the Differentiation of Sensory Hair Cells in the Development of Lateral Line Neuromasts

**FIGURE 3:**
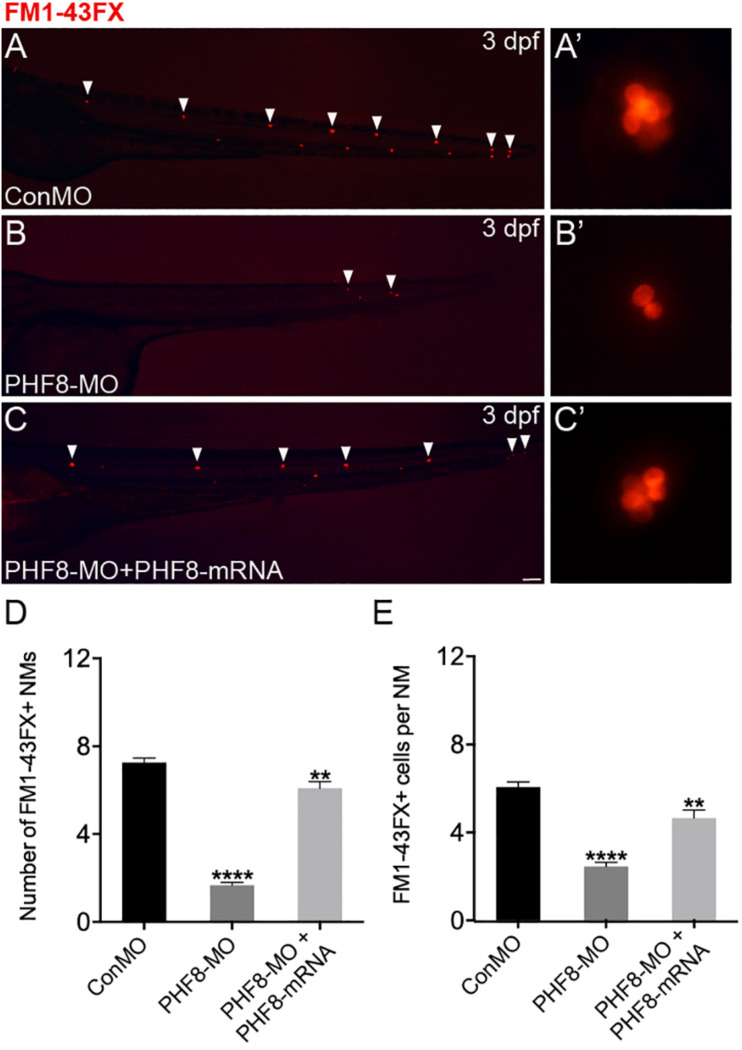
Analysis of neuromast hair cells in PHF8 morphant. **(A–C)** Embryos from different groups were stained with FM1-43FX at 3 dpf to detect functional neuromasts. **(A’–C’)** The enlarged FM1-43FX-positive neuromasts in controls **(A’)**, PHF8 morphants **(B’)**, and PHF8-MO co-injected with PHF8-mRNA embryos **(C’)** at 3 dpf. **(D)** The average number of FM1-43FX-positive neuromasts in controls (ConMO; *n* = 12 embryos), PHF8 morphants (PHF8-MO; *n* = 12 embryos), and PHF8-MO co-injected with PHF8-mRNA embryos (PHF8-MO + PHF8-mRNA; *n* = 12 embryos) at 3 dpf. **(E)** The average number of FM1-43FX-positive cells per neuromast (NM) in controls (ConMO; *n* = 20 neuromasts), PHF8 morphants (PHF8-MO; *n* = 20 neuromasts), and PHF8-MO co-injected with PHF8-mRNA embryos (PHF8-MO + PHF8-mRNA; *n* = 20 neuromasts) at 3 dpf. White arrowheads implicate the locations of the neuromasts on one side. **p < 0.01, ****p < 0.0001 vs. the ConMO group. Data are presented as means ± SEM. All pictures showed lateral view, anterior left. Scale bar, 100 μm. Neuromast, NM.

**FIGURE 4:**
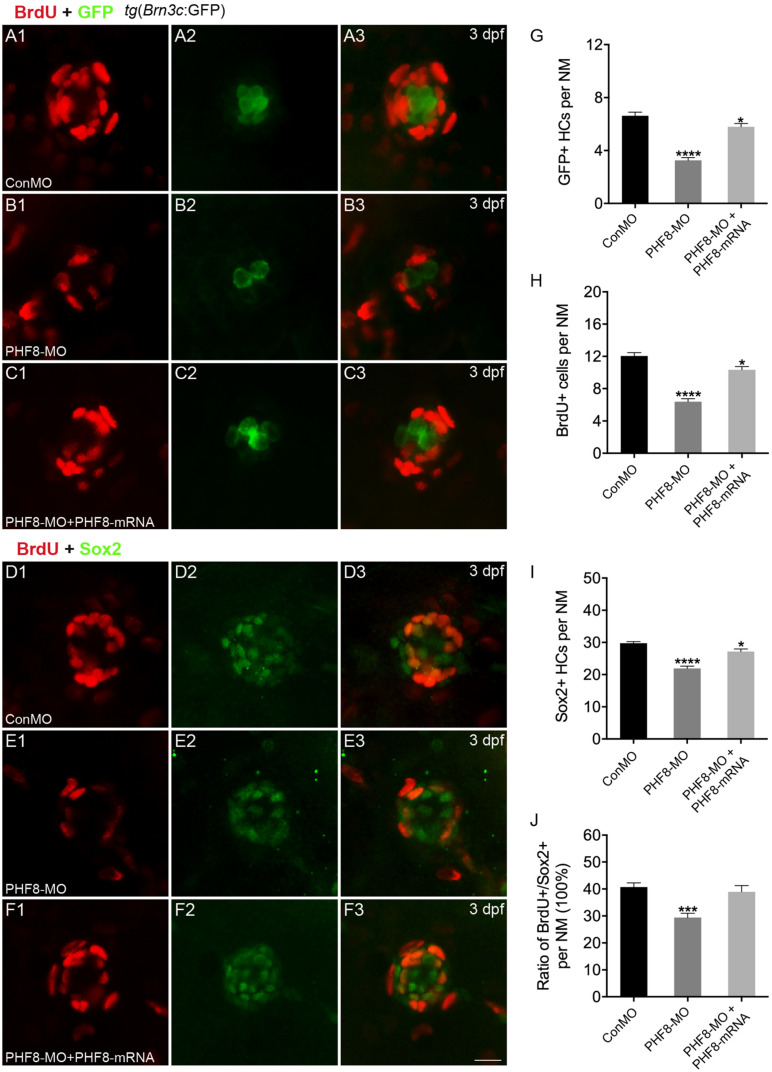
Analysis of neuromast proliferation in PHF8 morphant. **(A–C)** Representative images of GFP (green) and BrdU (red) staining of neuromasts from control (ConMO; **A**), PHF8 morphants (PHF8-MO; **B**), and PHF8-MO co-injected with PHF8-mRNA embryo (PHF8-MO + PHF8-mRNA; **C**) at 3 dpf. Hair cells were assessed with GFP visualization in transgenic line *tg*(*Brn3c*:GFP). **(D–F)** Representative images of supporting cell marker Sox2 (green) and BrdU (red) staining of neuromasts from control (ConMO; **D**), PHF8 morphants (PHF8-MO; **E**), and PHF8-MO co-injected with PHF8-mRNA embryo (PHF8-MO + PHF8-mRNA; **F**) at 3 dpf. **(G–J)** The average number of GFP-positive hair cells **(G)**, BrdU-positive cells **(H)**, Sox2-positive supporting cells **(I)**, and the ratio of BrdU + /Sox2 + cells per neuromast (NM) in control (ConMO), PHF8 morphants (PHF8-MO), and PHF8-MO co-injected with PHF8-mRNA embryo (PHF8-MO + PHF8-mRNA) at 3 dpf. *n* = 24 neuromasts per group, *p < 0.05, ***p < 0.001, ****p < 0.0001 vs. the ConMO group. Statistics are presented as means ± SEM. Scale bar, 10 μm. Neuromast, NM.

Based on the localization pattern of PHF8 in the neuromast cells, we assessed whether the hair cell number was changed in PHF8 morphants. Hair cell dyed with FM1-43FX was utilized to inspect the effect of PHF8 on the formation and deposition of hair cells and neuromasts in the lateral lines on the surface of the zebrafish body. As illustrated in Figure 3, the number of neuromasts labeled with FM1-43FX was remarkably decreased in 3 dpf PHF8 morphant. FM1-43FX is labeled on hair cells by traversing the mechanosensitive channels, thereby providing an index of mechanotransduction (Meyers et al., 2003). Thus, only functional hair cells with high activity could be dyed. It is possible that the normal number of hair cells are created in the morphants, but not successfully differentiated into fully mature hair cells; hence, FM1-43FX dying did not succeed in identifying these cells. To further examine whether PHF8 influenced the hair cells of neuromast, the transgenic zebrafish lines with GFP expression in the hair cells [*tg*(*Brn3c*:GFP)] were utilized (Xiao et al., 2005). At 3 dpf, *in vivo* imaging of transgenic embryos showed that the average number of GFP-positive hair cells in the neuromasts of control embryos at 3 dpf was 6.3 ± 0.19, which decreased to an average of 3.2 ± 0.18 hair cells in the PHF8 morphants (Figure 4).

To investigate whether cell proliferation inhibition is the mechanism underlying the decreased number of hair cells in neuromasts upon knockdown of PHF8, we co-stained PHF8 morphants with anti-BrdU, detecting proliferative cells and anti-GFP antibodies or anti-Sox2, a specific supporting cell marker. As suggested in Figure 4, BrdU labeling and the ratio of BrdU + /Sox2 + were significantly decreased in the neuromast of PHF8 morphants as compared to the controls at 3 dpf. Taken together, these data proposed the critical role of PHF8 in neuromast formation.

### Knockdown of PHF8 Disrupts Signaling Pathways Required for pLL Primordium Migration

**FIGURE 5:**
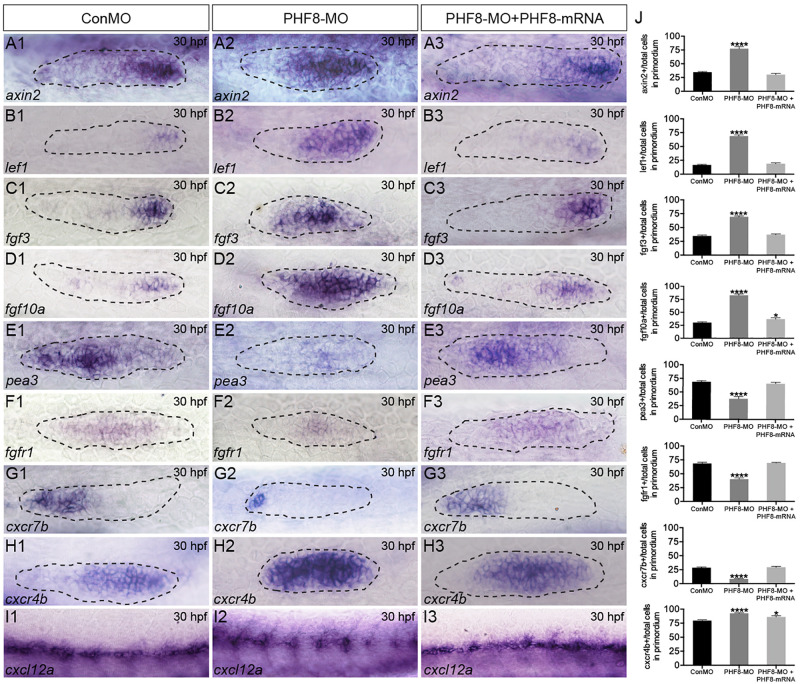
Analysis of expression of genes of Wnt and FGF signaling pathways, as well as *cxcl12/cxc7b/cxcr4b*. **(A–I)** WISH was utilized to examine the expression of *axin2* **(A)**, *lef1* **(B)**, *fgf3* **(C)**, *fgf10a* **(D)**, *pea3* **(E)**, *fgfr1* **(F)**, *cxcr7b* **(G)**, *cxcr4b* **(H)**, and *cxcl12* **(I)** at 30 hpf. **(J)** The ratio of *axin2*, *lef1*, *fgf3*, *fgf10a*, *pea3*, *fgfr1*, *cxcr7b*, and *cxcr4b*-positive cells to total cells in the primordia of controls (ConMO; *n* = 10 embryos for *axin2*, 10 embryos for *lef1*, 12 embryos for *fgf3*, 12 embryos for *fgf10a*, 11 embryos for *pea3*, 9 embryos for *fgfr1*, 10 embryos for *cxcr7b*, 12 embryos for *cxcr4b*), PHF8 morphants (PHF8-MO; *n* = 10 embryos for *axin2*, 10 embryos for *lef1*, 14 embryos for *fgf3*, 17 embryos for *fgf10a*, 9 embryos for *pea3*, 9 embryos for *fgfr1*, 15 embryos for *cxcr7b*, 17 embryos for *cxcr4b*), and PHF8-MO co-injected with PHF8-mRNA embryo (PHF8-MO + PHF8-mRNA; *n* = 10 embryos for *axin2*, 10 embryos for *lef1*, 10 embryos for *fgf3*, 9 embryos for *fgf10a*, 9 embryos for *pea3*, 10 embryos for *fgfr1*, 12 embryos for *cxcr7b*, 12 embryos for *cxcr4b*). Data are expressed as mean ± SEM. *p < 0.05, ****p < 0.0001. All optical appearances elucidate lateral visions; anterior is on the left. The primordium is delineated with a dashed line.

To identify whether the NM phenotype under observation in PHF8 morphants was induced by the abnormality of Wnt and FGF activity, the expression levels of Wnt and FGF signaling-related genes were examined in embryos. By 30 hpf, Wnt signaling-dependent expression of *axin2* and *lef1* was confined to a leading zone in control embryos; however, in PHF8 morphants, the expression of both genes extended into the trailing domain (Figures 5A,B). As the Wnt signaling pathway was activated, it stimulated the expression of *fgf3* and *fgf10a*. The expression of both genes was observed in the prominent domain in the control pLL primordia (Figures 5C,D). Conversely, the loss of PHF8 function increased the expressive ability of *fgf3* and *fgf10a* in the pLL primordia (Figures 5C,D).

FGF signaling is deemed necessary for both appropriate rosette formation within the migrating primordium and the deposition of the pLL neuromasts (Nechiporuk and Raible, 2008). Next, to examine whether FGF signaling was influenced in the PHF8 morphants, the expression of *pea3*, a downstream target of FGF signaling, was evaluated and found to be normal in the control pLL primordium (Figure 5E; Lecaudey et al., 2008; Nechiporuk and Raible, 2008). In contrast, loss of PHF8 function restricted *pea3* expression in the primordium (Figure 5E). Next, the transcript that encodes FGF receptor, *fgfr1*, was examined in the control pLL primordium (Figure 5F; Lecaudey et al., 2008; Nechiporuk and Raible, 2008). The gene expression was decreased in the pLL primordium of PHF8 morphants (Figure 5F) accompanied by a decrease in the expression of FGF signaling-reliant, *pea3*. These outcomes suggested that the observed NM phenotype in PHF8 morphants is associated with the defect in FGF signaling centers’ sequential formation and progressive restriction of the leading Wnt signaling.

The directional migration of the pLL primordium along the myoseptum is also regulated by *cxc7b/cxcr4b/cxcl12* expression (David et al., 2002; Li et al., 2004) in the migrating primordium. *Cxcr7b* expression, conventionally perceived in the trailing end of the immigrating pLL primordium and in the newly deposited neuromast cells in controlling embryos, was remarkably declined in the primordium of the PHF8 morphant embryos (Figure 5G). Conversely, *cxcr4b* was restricted to the dominant domain by 30 hpf in controlling embryos and expressed abundantly in PHF8 morphants (Figure 5H). Moreover, control embryos showed continuous *cxcl12a* expression along the horizontal myoseptum, while PHF8 morphants displayed impaired and discontinuous *cxcl12a* expression (Figure 5I).

### Knockdown of PHF8 Leads to Severe Otic Vesicle Deficiencies During Embryogenesis

**FIGURE 6:**
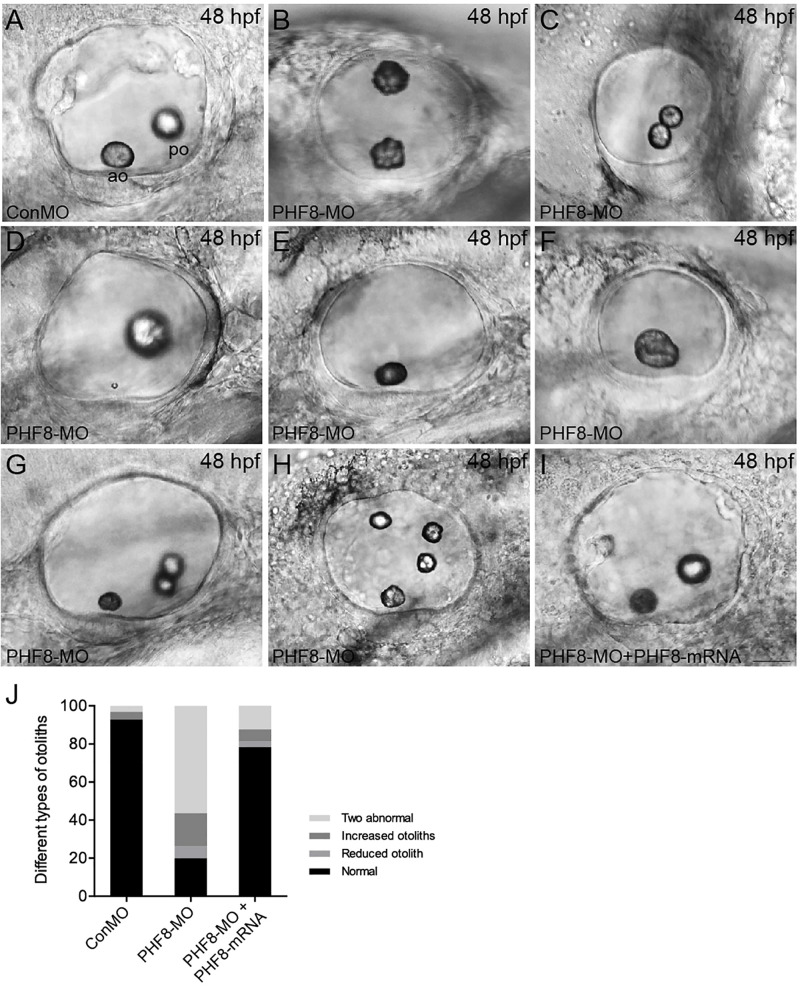
Analysis of otic vesicle formation in PHF8 morphant. **(A–I)** The overall morphology of otic vesicles in control embryos (ConMO; **A**), PHF8 morphants (PHF8-MO; **B–H**), and PHF8-MO co-injected with PHF8-mRNA embryos (PHF8-MO + PHF8-mRNA; **I**) at 48 hpf. According to the otolith phenotypes, zebrafish PHF8 morphants were divided into types as follows: the 2 abnormal otolith groups (**B**, misplaced; **C**, fused; **D**, tiny); the 1 otolith group **(E,F)**; and the multiple otolith group **(G,H)**. **(J)** Statistical results of different kinds of malformed otoliths (*n* = 125 for ConMO embryos, *n* = 126 for PHF8 morphants, and *n* = 75 for PHF8-MO + PHF8-mRNA embryos). The positions of the anterior otolith (ao) and posterior otolith (po) are implied. Optical appearances elucidate lateral visions; anterior is on the left. Scale bar, 25 μm.

Since the *PHF8* gene is expressed in the otic vesicle, its role in developing the otic vesicle in the zebrafish embryo was analyzed. In contrast to Con-MO-injected embryos (Figure 6A), the PHF8 morphants showed apparent otic defects (Figures 6B–H,J). The phenotype analysis of otoliths showed that the otolith formed in the majority of PHF8 morphants, but otoliths were mislocalized (Figure 6B), and under certain circumstances, odd-numbered otoliths were formed, and the morphants with three or four otoliths are displayed in Figures 6G,H. The reduced (Figures 6E,F), fused (Figure 6C), or abnormal size otoliths (Figure 6D) were also under observation in PHF8 morphants. To determine whether or not PHF8 is required at the earliest stages of otic placode development, we examined the expression of early placode marker *pax2a* and hindbrain patterning genes (*fgf3* and *fgf8*) at 12 hpf. Herein, we found that PHF8-MO injection did not affect these genes (Supplementary Figure 2). This phenomenon suggested that the otic vesicle deficiencies in PHF8 morphants might not be due to defects in the earliest stages of otic placode induction.

**FIGURE 7:**
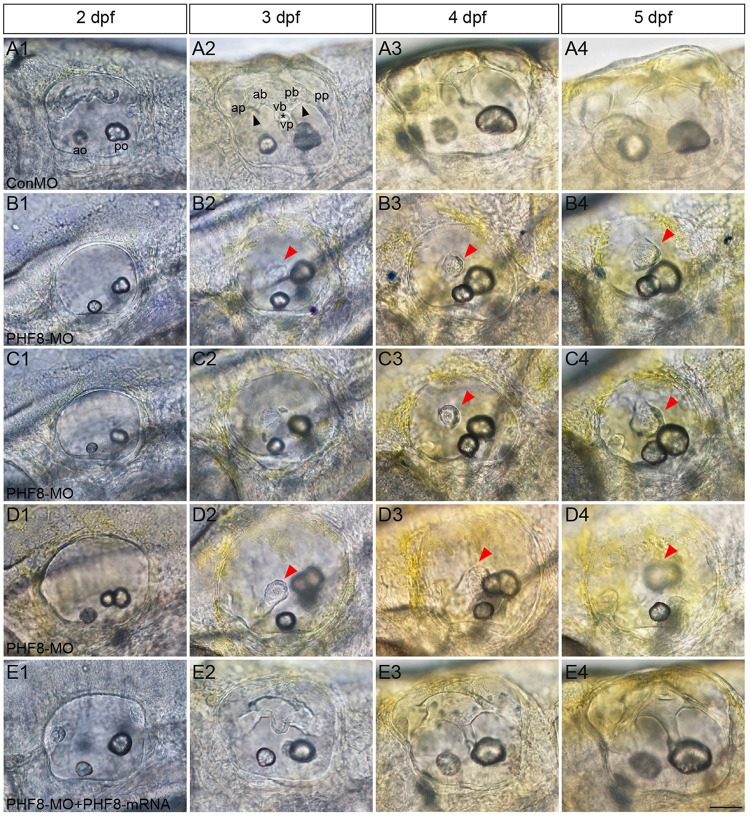
Analysis of semicircular canal formation in PHF8 morphant. **(A–E)** The overall morphology of semicircular canal in control embryos (ConMO; **A**), PHF8 morphants (PHF8-MO; **B–D**), and PHF8-MO co-injected with PHF8-mRNA embryos (PHF8-MO + PHF8-mRNA; **E**) at 2 dpf **(A1–E1)**, 3 dpf **(A2–E2)**, 4 dpf **(A3–E3)**, and 5 dpf **(A4–E4)**. Black arrowheads indicate the junction of the anterior protrusion (ap) and the anterior bulge (ab) and the junction of the posterior bulge (pb) and posterior protrusion (pp). Asterisks point out the junction between the ventral bulge (vb) and ventral protrusion (vp). Red arrowheads mark disrupted projections. The locations of the anterior otolith (ao) and posterior otolith (po) are implied. All pictures are lateral views with the anterior to the left and the dorsal side up. Scale bar, 50 μm.

Semicircular canal formation commences at 2 dpf by forming epithelial projections that invaginate from the walls of the otic vesicle wall toward its lumen (Waterman and Bell, 1984). Compared to controls at 2 dpf (Figure 7A1), the lateral protrusions in PHF8 otic vesicles seem to be standard size despite the smaller size of the otic vesicle (Figures 7B1–D1). At 3 dpf, the progressing semicircular canal protrusions of controls merged in the ear lumen, forming a specific cruciform look when viewed from the lateral side (Figure 7A2); however, the canals of the phenotypically severe PHF8 otic vesicles were morphologically irregular (Figures 7B2–D2). By 4 dpf, controls had normal outgrowth of the semicircular canals in the inner ear (Figure 7A3). In PHF8 morphants, in the same phase, the protrusions did not show a cruciform shape but were disordered and collapsible within the otic vesicle lumen (Figures 7B3–D3). In order to verify that the inner ear defect observed in morphants was explicitly due to the disrupted PHF8 function, we injected *PHF8*-mRNA in combination with PHF8-MO. Consequently, the majority of the co-injected embryos displayed normal inner ear (Figure 6I and Figures 7E1–E4).

**FIGURE 8:**
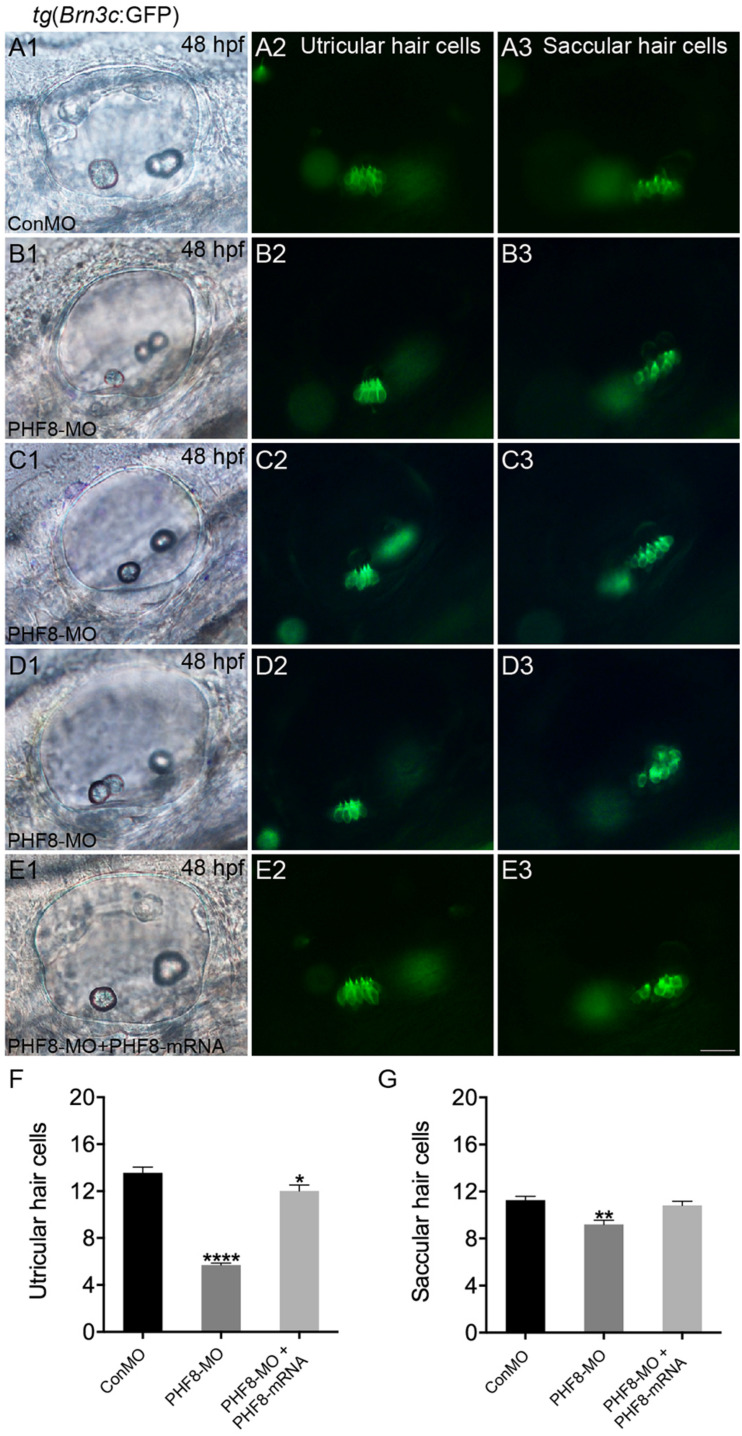
The requirement of PHF8 for hair cells in the inner ear. **(A–D)** Representative images of GFP (green) in the utricle and saccule of control embryo (ConMO; **A1–A3**), PHF8 morphant (PHF8-MO; **B1–B3**,**C1–C3**,**D1–D3**), and PHF8-MO co-injected with PHF8-mRNA embryos (PHF8-MO + PHF8-mRNA; **E1–E3**) at 48 hpf. Hair cells were assessed with GFP visualization in transgenic line *tg*(*Brn3c*:GFP). **(F–G)** Quantification of the numbers of hair cells in the utricle and saccule of the inner ear for each experimental condition at 48 hpf. *p < 0.05, **p < 0.01, ****p < 0.0001 vs. the ConMO group. Data are presented as means ± SEM (*n* = 16 embryos, per each). Optical appearances elucidate lateral visions; anterior is on the left. Scale bar, 25 μm.

The otic deficiency in PHF8 morphants implied a putative function for PHF8 in the progression of hair cells. In order to examine if the knockdown of PHF8 is detrimental to hair cell formation in the inner ear, transgenic embryos were injected with PHF8-MO, which expressed *Brn3c*:GFP. Consequently, hair cell production was not obstructed but declined in PHF8 morphants (Figure 8). Moreover, knockdown of PHF8 led to a remarkable decrease in the number of hair cells in both utricular and saccular maculae (Figures 8B–D). At 48 hpf, the control utricle contained 13.6 ± 0.47 hair cells (Figures 8A,F), whereas 5.7 ± 0.18 hair cells were discovered in the utricle of PHF8 morphants. Also, the average number of hair cells in saccules (11.3 ± 0.35 in controls; 9.2 ± 0.37 in PHF8 morphants, Figure 8G) declined. In order to confirm whether the effects of PHF8-MO on hair cell development could be rescued, we co-injected PHF8-MO with *PHF8*-mRNA and found that such embryos produced more GFP-positive hair cells than PHF8 morphants (Figures 8E1–E3,F,G). The statistical analysis implied that PHF8 regulates the formation of hair cells in the inner ear.

## Discussion

The present study reported that PHF8 is significant for developing the pLL and inner ear in zebrafish. PHF8 knockdown impaired normal sensory responses, disrupted pLL primordium migration, decreased neuromast deposition, and affected the inner ear’s development. In addition to *cxcr4b* and *cxcr7b* expression, the influence of PHF8 on pLL primordium arises partially from Wnt and FGF signaling pathways’ dysregulation. For the first time, this study proved that PHF8 is critical for the development of auditory organs.

We found that PHF8 is highly expressed in the primordium of pLL and in the otic vesicle, which implicates that it may play a role in the hearing procedure. To further investigate the function of PHF8, PHF8-MO zebrafish model was established, and we discovered that the migration of pLL and the deposition of neuromasts were remarkably influenced in the PHF8-knockdown fish. The use of MOs in studying the development is often accompanied by off-target effects that cause developmental defects (Eisen and Smith, 2008). In the present study, we addressed this issue by PHF8- and p53-MO co-injection experiments. Consistent with this result in PHF8-knockdown experiments, the number of neuromasts in PHF8- and p53-MO co-injected embryos also decreased significantly. In addition, the outcomes in the RNA rescue tests on the recovery of neuromast numbers confirmed the unique effects of PHF8-MO. These results also indicated the role of PHF8 in the progress of hair cell differentiation in the neuromasts of pLL. Nonetheless, little cell proliferation was observed in the neuromast of PHF8 morphants at 3 dpf, which implicated that the decrease in the number of hair cells was partial because of the inhibition of proliferation in the neuromasts.

In zebrafish, the migration of the primordium of the pLL to the tail begins at 20–22 hpf from the pLL placode, situated at the hindbrain, just posterior to the otic placode (Dambly-Chaudiere et al., 2003). Then, the primordium migrates caudally, followed by the formation of proneuromasts that differentiate in the following few hours (Gompel et al., 2001). Two reciprocally antagonistic signaling centers primarily control the progress: the Wnt signaling center in the prominent domain of migrating primordia and the FGF signaling pathway in the trailing domain (Aman and Piotrowski, 2008; Ma and Raible, 2009). The foundation of polarized Wnt and FGF signaling systems harmonizes the primordium’s morphogenesis and migration (Nogare and Chitnis, 2017). The FGF signaling pathway activates at the primordium’s trailing cells and is crucial for the production of proneuromasts from the primordium. On the other hand, Wnt signaling is a predominant pathway at the leading cells and modulates such modeling to some extent by regulating FGF signaling (Lecaudey et al., 2008; Nechiporuk and Raible, 2008). As group migration commences and the primordium changes residence, the originally extensive Wnt active zone shrinks closer to the dominant brink. Additional FGF signaling centers are formed sequentially and associated with the formation of proneuromasts (Nogare and Chitnis, 2017).

An active Wnt pathway is essential for the expression of FGF ligands, *fgf3* and *fgf10a*, in the leading zone of the primordium, and the overstimulation of Wnt or attenuation of FGF signaling leads to abnormal primordium patterns, such as the expansion of the leading zone genes (*fgf3*, *fgf10a*, *lef1*, and *axin2*) and the loss of a downstream target of FGF signaling gene (*pea3*), suggesting a negative-feedback regulation (Aman and Piotrowski, 2008; Nechiporuk and Raible, 2008). In order to investigate the molecular mechanism underlying the decreased neuromast deposition and disturbed migration of the pLL primordium, we conducted WISH with probes against Wnt and FGF signaling pathways. The current studies revealed that knockdown of PHF8 significantly increased the expression of *fgf3*, *fgf10a*, *lef1*, and *axin2* and decreased the expression of FGF target genes (*pea3* and *fgfr1*), suggesting that pLL phenotypes in PHF8 morphants are associated with expanding Wnt signaling to a broad prominent domain in the primordium and blocking FGF signaling at the trailing end of the pLL primordium. Reportedly, blocking FGF signaling leads to a disordered primordium after the deposition of a single neuromast and a ceased primordium migration, due to which attenuated FGF signaling is ascribed in the failure of neuromast formation and primordium migration in PHF8 morphants.

In addition, *cxcl12/cxc7b/cxcr4b* is another vital guiding signaling system for the directional migration of the pLL primordium (David et al., 2002; Li et al., 2004). Wnt and FGF signaling pathways regulate the primordium via the expression of the *cxcr4b* and *cxcr7b* (Aman and Piotrowski, 2008). The inhibition of the pharmacological FGF pathway by SU5402 leads to the loss of the ectopic expression of *cxcr4b* and *cxcr7b*, which influences the migration of the pLL primordium (Aman and Piotrowski, 2008). The current results showed that in PHF8 morphants, the *cxcr4b* zone is enlarged, and the size of the cxcr7b domain is remarkably decreased, which confirmed that the defect in expanding leading Wnt signaling and restricting FGF signaling system in PHF8 morphants is defective with respect to the expression of polarized chemokine receptor.

Furthermore, we studied the function of PHF8 during the development of the inner ear. Placode induction and vesicle cavitation seemed to be of normal condition, but the otic vesicle’s morphogenesis was influenced by defective PHF8. The dissimilarities were detected in the phenotypes of the inner ear between PHF8 morphants and controls. The knockdown of PHF8 resulted in abnormal otolith number, failure of otoliths to grow at appropriate positions, and decreased number of hair cells in the maculae of the inner ear. The PHF8 morphants demonstrated a formative abnormality of the semicircular canal with disrupted or even absent semicircular canal outgrowth.

PHF8 has been predominantly reported as a transcriptional activator due to its ability to demethylate multiple repressive histone modifications, including H3K9me1/2, H4K20me1, and H3K27me2 (Liu et al., 2010; Loenarz et al., 2010; Qi et al., 2010). Interestingly, PHF8 also binds to an active histone marker H3K4me3 through the *N*-terminal PHD domain and exhibits H3K4 demethylase activity (Qiu et al., 2010). A previous study indicated that PHF8 may also suppress the transcription of specific target genes, although the underlying mechanisms are still unknown (Wang et al., 2014). Recent data documented the involvement of the epigenetic regulators HDAC1 and HDAC3 in the development of zebrafish inner ear and lateral line system as well as the loss of HDAC1/3 function in the impaired formation of the inner ear and pLL (He et al., 2016a,b). In the present study, we found that PHF8 knockdown affects the zebrafish inner ear and the development of the lateral line system. At the same time, the abnormal activity of PHF8 is associated with aberrant gene expression. Given the colocalization and function of HDAC1/3 and PHF8 in zebrafish inner ear and lateral line system, understanding the precise molecular mechanisms underlying the crosstalk between these epigenetic enzymes in chromatin remodeling and regulation of gene transcription will reveal the regulation of disease progression and open novel therapeutic opportunities. Future studies with the genome-wide epigenetic regulation of genes in PHF8 mutant will help to uncover the potential epigenetic mechanisms.

In summary, this study provides evidence of the role of PHF8 in the development of zebrafish auditory organs. Additional studies are required to develop more in-depth insights into the molecular mechanisms and elucidating the role of PHF8 in organogenesis and deafness in the future.

## Data Availability Statement

The raw data supporting the conclusions of this article will be made available by the authors, without undue reservation.

## Ethics Statement

The animal study was reviewed and approved by the Institutional Animal Care and Use Committee of Xiamen University.

## Author Contributions

JH performed the experiment and drafted the manuscript. ZZ performed the experiments, interpreted and analyzed the data, and approved the final manuscript. XL, YH, and WS interpreted and analyzed the data and approved the final manuscript. CC conceived and designed the study, interpreted and analyzed the data, and approved the final manuscript. All authors contributed to the article and approved the submitted version.

## Conflict of Interest

The authors declare that the research was conducted in the absence of any commercial or financial relationships that could be construed as a potential conflict of interest.

## Supplementary Material

Supplementary Figure 1 **(A)** The effectiveness of PHF8-MO was detected by RT-PCR at 24 hpf. **(B)** Gross phenotypic morphology of control and PHF8 morphant embryo.

Supplementary Figure 2 Expression of *pax2*, *fgf3*, and *fgf8* in the zebrafish embryos at 12 hpf. **(A–F)** WISH was utilized to examine the expression of *pax2* **(A,B)**, *fgf3* **(C,D)**, and *fgf8* **(E,F)** at 12 hpf.
