# Supplementary figures and images for: Histone Demethylase PHF8 Is Required for the Development of the Zebrafish Inner Ear and Posterior Lateral Line
Source: Front Cell Dev Biol. 2020 Nov 23;8:566504. doi: 10.3389/fcell.2020.566504 (PMC7719749; doi:10.3389/fcell.2020.566504)

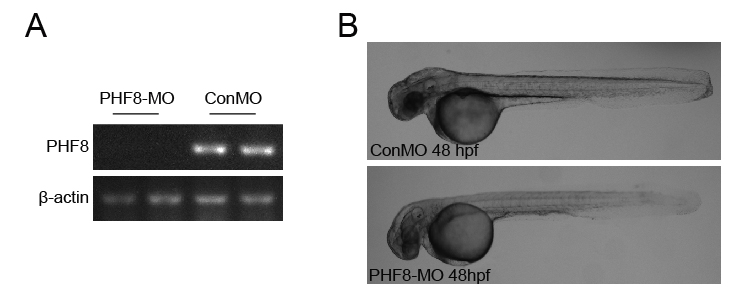

Supplement: Supplementary Figure 1 — (A) The effectiveness of PHF8-MO was detected by RT-PCR at 24 hpf. (B) Gross phenotypic morphology of control and PHF8 morphant embryo. [file Image_1.JPEG]

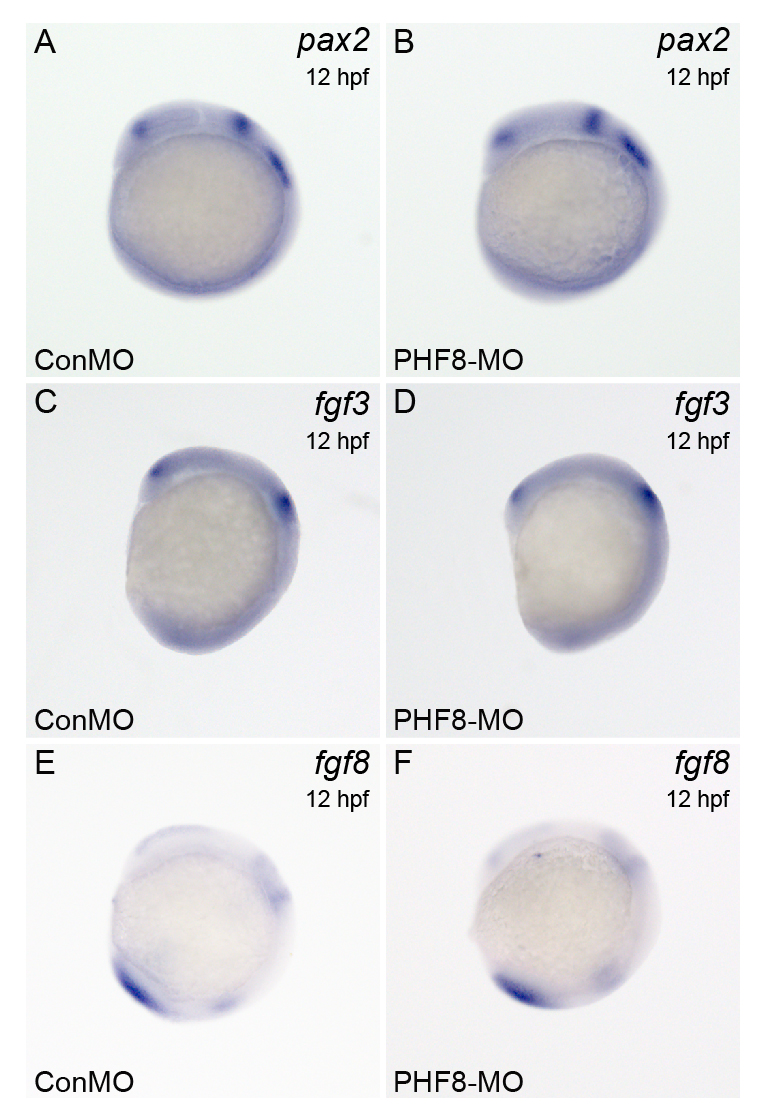

Supplement: Supplementary Figure 2 — Expression of pax2, fgf3, and fgf8 in the zebrafish embryos at 12 hpf. (A–F) WISH was utilized to examine the expression of pax2 (A,B), fgf3 (C,D), and fgf8 (E,F) at 12 hpf. [file Image_2.JPEG]
